# PATTERNS OF SOCIAL ENGAGEMENT AMONG OLDER ADULTS WITH MILD COGNITIVE IMPAIRMENT

**DOI:** 10.1093/geroni/igz038.418

**Published:** 2019-11-08

**Authors:** Takashi Amano, Nancy Morrow-Howell, Sojung Park

**Affiliations:** 1 Brown School, Washington University in St. Louis, St. Louis, Missouri, United States; 2 Washington University, St. Louis, Missouri, United States

## Abstract

Promoting engagement in social activities may be an intervention that prevents or delays cognitive impairment. Nevertheless, little is known about social engagement among people with mild cognitive impairment. We aim to examine patterns of social engagement among people with mild cognitive impairment and to assess whether factors under four domains of the WHO's ICF model (personal factors, environmental factors, body functions and structures, and health condition) are associated with patterns. Data were drawn from the 2010 Health and Retirement Study. The final sample comprised 1,227 people with Cognitive Impairment No Dementia (CIND). Latent class analysis and multinomial logistic regression were utilized. Three patterns of social engagement were identified: informal social engagement only, formal and informal social engagement, and low social engagement. At least one factor from each domain was associated with the probability of class membership. Our findings suggest that social engagement is heterogeneous among people with CIND and that some groups of people with CIND have possibilities of engaging in more social activities, especially in formal social activities. Results also indicate that providing informal social resources may be essential for social programs designed specifically for people with CIND to promote their formal social engagement. Future study is needed to examine possible differences in outcomes across groups with similar patterns of social engagement.

